# Modulating IL‐1R1/IL‐1RAP Signaling: A Dual Approach to Reducing Neuroinflammation and Tau Pathology

**DOI:** 10.1002/alz70855_105590

**Published:** 2025-12-24

**Authors:** Somayeh Dadras, Cristian Bologa, Tione Buranda, Kiran Bhaskar

**Affiliations:** ^1^ University of New Mexico, Albuquerque, NM, USA; ^2^ University of New Mexico, lbuquerque, NM, USA

## Abstract

**Background:**

Alzheimer's disease (AD) and related tauopathies are increasingly significant causes of death worldwide, yet no cure exists. IL‐1β‐induced inflammation plays a pivotal role in tau pathology, neurodegeneration, and cognitive decline in this group of diseases. When IL‐1β binds to its receptor, IL‐1 receptor 1 (IL‐1R1), it engages IL‐1 receptor accessory protein (IL‐1RAP), eventually leading to NF‐κB activation. IL‐1RAP is also a co‐receptor for IL‐1α, IL‐33, and IL‐36, all implicated in AD and neurodegeneration. Genome‐wide association Studies have linked *IL1RAP* to AD. Single nucleotide polymorphisms in this gene positively correlate with an increased risk of progressing from mild cognitive impairment to AD and greater brain atrophy. Despite evidence pointing to IL‐1RAP's involvement in AD/tauopathies, its precise role remains unclear. This study aims to elucidate the impact of IL‐1RAP depletion or inhibition, either genetically or through FDA‐approved pharmaceuticals, on neuroinflammation and tau pathology.

**Methods:**

We generated IL‐1RAP myeloid‐cell restricted conditional knockout mice (mcKO) to assess the impact of myeloid cell‐specific deletion of IL‐1RAP on the AD phenotype via crossing them to the PS19 mouse model of tauopathy. Western blotting and immunohistochemistry (IHC) were used to assess tau pathology. Levels of inflammatory cytokines were examined via Meso Scale Discovery (MSD). To identify pharmaceutical inhibitors of IL‐1R1/IL‐1RAP, we screened the Prestwick Chemical Library using HEK‐Blue IL‐1β cells and after the addition of compounds and IL‐1β and a 24‐hour incubation, measured the secreted embryonic alkaline phosphatase in the supernatant.

**Results:**

Analysis of hippocampal lysates from PS19 and PS19/IL‐1RAP mcKO mice using Western blot and IHC for pathological tau and MSD for inflammatory cytokines revealed a significant reduction in tau pathology and proinflammatory cytokines in the knockout mice. Behavioral tests are currently underway to assess whether this reduced inflammation and tau pathology in the PS19/IL‐1RAP mcKO mice improves cognition. Through high‐throughput screening, we identified 20 small molecule drugs significantly inhibiting the IL‐1R1/IL‐1RAP signaling axis. These compounds are currently undergoing validation.

**Conclusions:**

Our results indicate that inhibiting the IL‐1R1/IL‐1RAP signaling axis is a promising approach to reducing neuroinflammation and tau pathology. This approach could also be effective for treating other inflammatory diseases.

